# A case of surgically treated recurrent spontaneous pneumothorax during pregnancy

**DOI:** 10.1093/omcr/omag109

**Published:** 2026-06-21

**Authors:** Žymantas Jagelavičius, Jekaterina Strelčenko, Jelena Volochovič, Edvardas Žurauskas, Ričardas Janilionis

**Affiliations:** Clinic of Chest Diseases, Immunology, and Allergology, Institute of Clinical Medicine, Vilnius University Faculty of Medicine, Santariskiu 2, Vilnius LT-08406, Lithuania; Vilnius University Faculty of Medicine, Santariskiu 2, Vilnius LT-08406, Lithuania; Clinic of Obstetrics and Gynecology, Institute of Clinical Medicine, Vilnius University Faculty of Medicine, Santariskiu 2, Vilnius LT-08406, Lithuania; Department of Pathology and Forensic Medicine, Institute of Biomedical Sciences, Vilnius University Faculty of Medicine, P. Baublio 5, Vilnius LT-08406, Lithuania; Clinic of Chest Diseases, Immunology, and Allergology, Institute of Clinical Medicine, Vilnius University Faculty of Medicine, Santariskiu 2, Vilnius LT-08406, Lithuania

**Keywords:** spontaneous pneumothorax, pregnancy, recurrent pneumothorax, thoracoscopic surgery

## Abstract

Spontaneous pneumothorax in pregnancy is a very uncommon condition, with fewer than 100 cases described in the English literature. This case report describes a pregnant woman who experienced two episodes of primary spontaneous pneumothorax during pregnancy. A 31-year-old, Caucasian pregnant woman (P2 G3 A1, gestational age 17^+0^ weeks), conceived naturally, body mass index 28, a former smoker, arrived at the hospital with dyspnea and chest pain. Pneumothorax was detected and managed with a chest tube. However, three days after discharge, pneumothorax reoccurred. Due to a persistent air leak, video-assisted thoracoscopic surgery was performed. Histological examination revealed enlarged airspaces without any signs of specific lung pathology. Postoperative recovery was uneventful, and the patient delivered a healthy newborn without complications. This case highlights the importance of multidisciplinary, individualized diagnostic and treatment options in pregnant patients with primary spontaneous pneumothorax to prevent misdiagnosis and ensure optimal outcomes for both mother and fetus.

## Introduction

Primary spontaneous pneumothorax is the spontaneous occurrence of air in the pleural cavity in patients without clinically or radiologically known underlying lung disease. It is more common in tall, thin, and young men [1]. Spontaneous pneumothorax in pregnancy is a very uncommon condition, with fewer than 100 cases described in the literature [2]. In this study, we present a clinical case of two episodes of primary spontaneous pneumothorax in a pregnant woman.

## Case report

A 31-year-old Caucasian pregnant woman (P2 G3 A1, gestational age 17^+0^ weeks) who conceived naturally, with a body mass index of 28 and a smoking history until pregnancy (10 pack-years), without any known previous diseases, experienced sharp pain in the right chest. A chest X-ray revealed a right-sided pneumothorax (Fig. 1). The patient was admitted to the hospital, and a chest tube was inserted. The patient improved, the lung expanded, and the chest tube was removed. There were no clinical or ultrasound signs of residual pneumothorax. The fetus was found to be well, so the patient was discharged.

**Figure 1:**
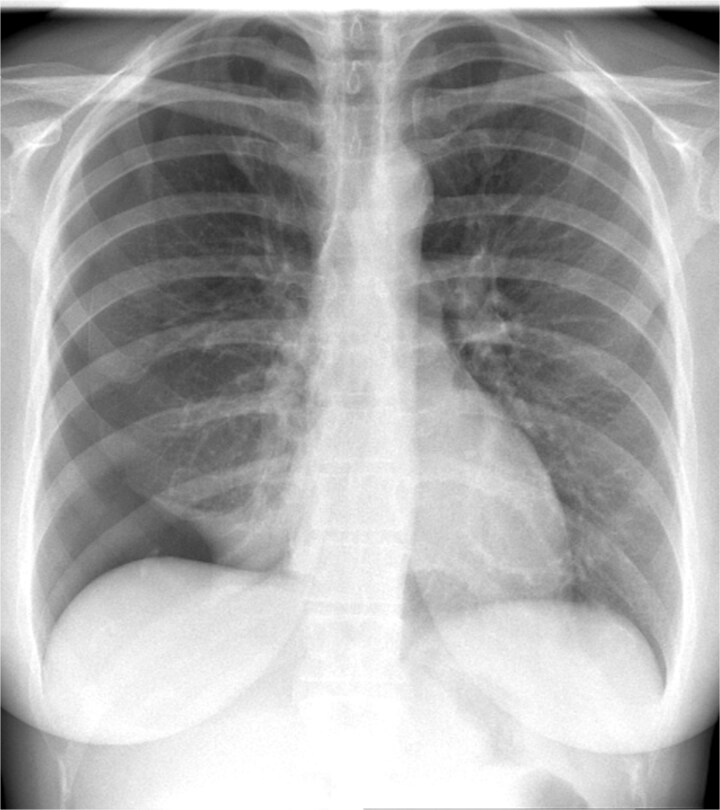
Air in the right pleural cavity and collapsed right lung—The first episode of the spontaneous pneumothorax.

Three days later, she again experienced sudden, sharp pain on the right side of her chest. On auscultation, weakened breathing sounds were observed on the right. A chest X-ray revealed recurrent pneumothorax. Due to the existing pregnancy, it was decided to insert a chest tube again. However, a persistent air leak was present, and the lung did not fully expand. The situation was discussed among the obstetrician-gynecologist, thoracic surgeon, and anesthesiologist, and surgery was decided upon. A right video-assisted thoracoscopic surgery (VATS) was performed. Some adhesions and blebs (up to 1.5 cm) were found in the apex of the lung (Fig. 2), and no pathological changes in the other parts of the lung or the diaphragm were noticed. Wedge resection of the lung and apical parietal pleurectomy was performed. The postoperative period was uneventful, and on the seventh postoperative day, the patient was discharged with the right lung fully expanded. The fetus was developing normally at 21^+1^ weeks of gestation. Histological examination revealed dilated airspaces under the visceral pleura. Immunohistochemical staining for microphthalmia-associated transcription factor (MiTF), estrogen receptor (ER), CD34, alpha-smooth muscle type actin (SMA), and HMB-45 was negative (Fig. 3). Further pregnancy and fetal development were normal. At 39^+4^ weeks of pregnancy, the patient spontaneously began regular labor. A male newborn, weighing 3300 g and 54 cm tall, was born via natural delivery. The Apgar score was 9/10. The postpartum period was uneventful for the mother and the newborn.

**Figure 2:**
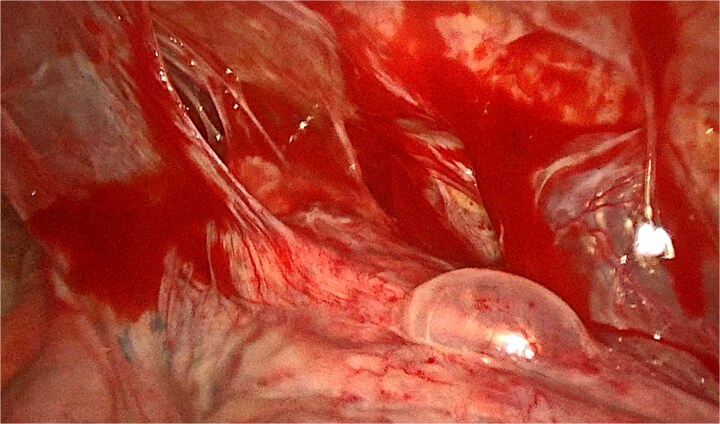
Right-sided video-assisted thoracic surgery: Adhesions and blebs on the surface of the upper lobe.

**Figure 3:**
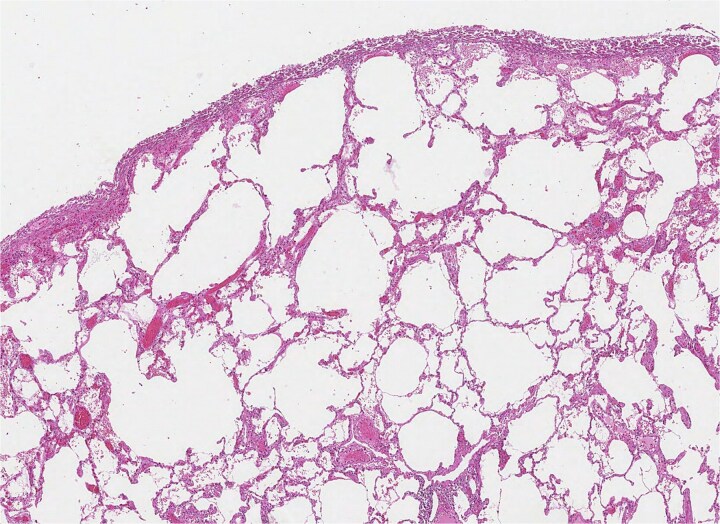
Histological examination. HEx4. Dilated air spaces under the visceral pleura. Immunohistochemical staining for MiTF, ER, CD34, SMA, and HMB-45 was negative.

Abdominal and lower chest CT scan 10 months later revealed several small, thin-walled intrapulmonary cysts and a pathological mass in the abdominal cavity (Fig. 4). Unfortunately, the patient was lost to further follow-up.

**Figure 4:**
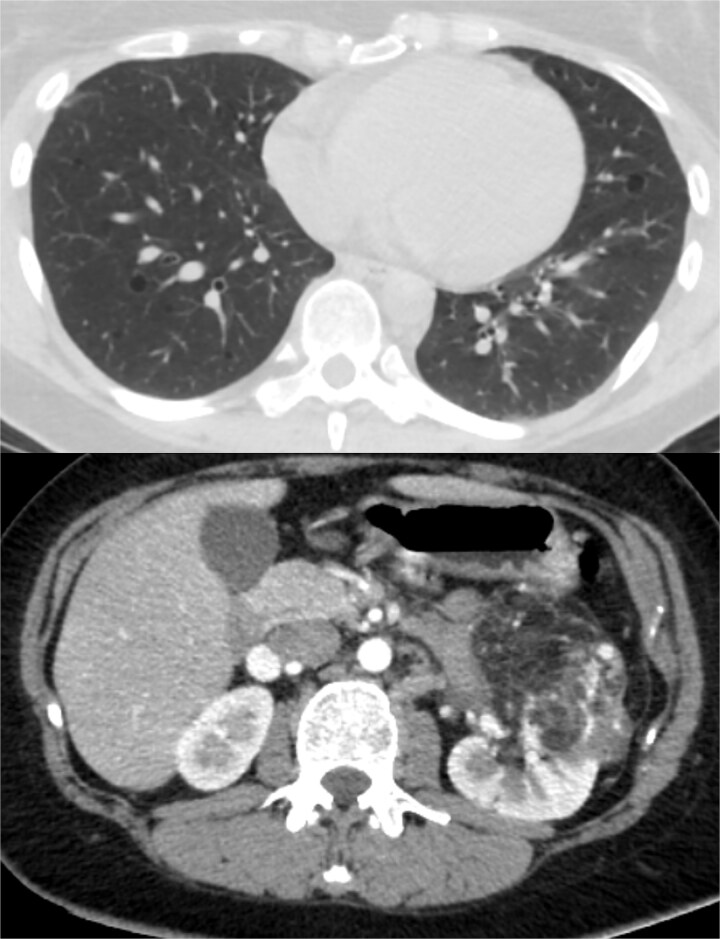
CT scan 10 months later: Small thin-walled intrapulmonary cysts and possible angiomyolipoma in the abdominal cavity.

## Discussion

Literature estimates that spontaneous pneumothorax occurs in 7–18 cases/100000 population/year in men and 1–6 cases/100000 population/year in women [1]. However, the exact prevalence of the disease during pregnancy is unknown due to the limited number of cases described in the literature and the possibility of underestimation or misdiagnosis [3, 4]. According to a recent systematic review, 47% of cases occur in the first two trimesters of pregnancy, 36% in the third trimester, and the remainder during labor or the postpartum period [2]. In our case, episodes occurred in the second trimester of pregnancy. When a pneumothorax recurs very quickly, it is very difficult to distinguish it from a possible ongoing, very slow air leak as a continuation of the first episode.

The increased demand for oxygen during pregnancy poses some risks to the mother and fetus [3, 4]. The leading cause of primary spontaneous pneumothorax is the rupture of blebs or bullae on the surface of the lung, which is also a case in pregnant patients [4]. Cystic lung diseases, including lymphangioleiomyomatosis (LAM), pulmonary Langerhans cell histiocytosis, and Birt-Hogg-Dubé syndrome, may also cause pneumothorax. LAM occurs almost exclusively in women of childbearing potential. It is a rare disorder associated with changes in the tuberous sclerosis complex genes, leading to gradual, low-grade changes in the lung parenchyma. In our case, histologically, neither LAM nodules nor any other signs of this disease were found. However, small, thin-walled lung cysts and an abdominal angiomyolipoma suggest that the case is most likely LAM.

Spontaneous pneumothoraces can rarely be associated with pregnancy. Wright et al. described a very rare case of trophoblastic disease and spontaneous pneumothorax presented in the 37^th^ week of pregnancy [5]. Spontaneous pneumothorax can also occur during labor, usually due to repeated Valsalva maneuvers, which is known as Hamman syndrome [6]. Hyperemesis gravidarum has also been described as an unusual cause of pneumomediastinum and pneumothorax [7].

A chest X-ray is the main diagnostic tool for pneumothorax. Chest X-ray with abdominal protection is a reasonably safe [8]. The fetus is most sensitive to radiation during organogenesis (2–7 weeks after conception) and the early fetal period (8–15 weeks after conception) [9]. Therefore, it is imperative to follow the requirements of radiation protection for pregnant women.

The treatment generally does not differ from that for spontaneous pneumothorax. The essential difference between the two situations is that the pregnant patient and the fetus may poorly tolerate respiratory failure caused by pneumothorax [2, 8]. The second trimester of pregnancy is considered optimal for surgical intervention, as organogenesis is already complete, and the risk of preterm birth is relatively lower than in the later stages of pregnancy [2, 3]. For recurrent pneumothorax, VATS surgery is performed with or without lung resection, combined with mechanical pleurodesis [2]. After the birth, a high-resolution chest CT should be performed, and the woman should be consulted by a pulmonologist. Without follow-up and further testing, we cannot be sure about cystic lung disease.

Spontaneous pneumothorax is usually not a contraindication to natural childbirth. However, Valsalva maneuvers may increase the risk of recurrence of pneumothorax [10]. With pleural drainage alone, the risk of recurrence is high, at approximately 30%–40% [3]. However, after surgical treatment, the risk of recurrence is minimal.

Spontaneous pneumothorax in pregnancy is a very rare but potentially dangerous condition for both the woman and the fetus. It is important to consider specific causes of spontaneous pneumothorax in pregnant women.
